# Use of non-steroidal anti-inflammatory drugs and risk of breast cancer: The Spanish Multi-Case-control (MCC) study

**DOI:** 10.1186/s12885-016-2692-4

**Published:** 2016-08-20

**Authors:** Trinidad Dierssen-Sotos, Inés Gómez-Acebo, María de Pedro, Beatriz Pérez-Gómez, Sonia Servitja, Víctor Moreno, Pilar Amiano, Tania Fernandez-Villa, Aurelio Barricarte, Adonina Tardon, Marian Diaz-Santos, Rosana Peiro-Perez, Rafael Marcos-Gragera, Virginia Lope, Esther Gracia-Lavedan, M. Henar Alonso, Maria Jesus Michelena-Echeveste, Andrés Garcia-Palomo, Marcela Guevara, Gemma Castaño-Vinyals, Nuria Aragonés, Manolis Kogevinas, Marina Pollán, Javier Llorca

**Affiliations:** 1 CIBER Epidemiologia y Salud Publica (CIBERESP), Madrid, Spain; 2 University of Cantabria – IDIVAL, Santander, Spain; 3 Department of Obstetrics and Gynecology, Nuevo Belén University Hospital, Madrid, Spain; 4 Cancer and Environmental Epidemiology Unit, National Center for Epidemiology, Carlos III Institute of Health, Madrid, Spain; 5 Cancer Epidemiology Research Group, Oncology and Hematology Area, IIS Puerta de Hierro (IDIPHIM), Madrid, Spain; 6 Servei d’Oncologia Mèdica, Hospital del Mar, Barcelona, Spain; 7 Cancer Research Program IMIM (Hospital del Mar Medical Research Institute), Barcelona, Spain; 8 Cancer Prevention and Control Program, Catalan Institute of Oncology-IDIBELL, and University of Barcelona, Barcelona, Spain; 9 Public Health Division of Gipuzkoa, Biodonostia Research Institute, San Sebastian, Spain; 10 Área de Medicina Preventiva y Salud Pública, Departamento de Ciencias Biomédicas, Universidad de León, León, España; 11 Grupo de Investigación en Interacciones Gen-Ambiente y Salud (GIIGAS), Universidad de León, León, España; 12 Navarra Public Health Institute, Pamplona, Spain; 13 Navarra Institute for Health Research (IdiSNA), Pamplona, Spain; 14 IUOPA, Universidad de Oviedo, Oviedo, Spain; 15 Universidad de Huelva, Huelva, Spain; 16 Centro de Investigación en Salud y Medio Ambiente (CYSMA), Huelva, Spain; 17 Area de Cáncer y Salud Pública, Fundación FISABIO- Salud Pública, Valencia, Spain; 18 Epidemiology Unit and Girona Cancer Registry, Oncology Coordination Plan, Department of Health, Autonomous Government of Catalonia and Descriptive Epidemiology, Genetics and Cancer Prevention Group [Girona Biomedical Research Institute (IdIBGi)], Catalan Institute of Oncology, Girona, Spain; 19 Centre for Research in Environmental Epidemiology (CREAL), Barcelona, Spain; 20 Universitat Pompeu Fabra (UPF), Barcelona, Spain; 21 Onkologikoa- Oncology Institute Gipuzkoa, San Sebastian, Spain; 22 Sección de Oncología, Complejo Asistencial Universitario de León, León, Spain; 23 IMIM (Hospital del Mar Medical Research Institute), Barcelona, Spain

**Keywords:** Breast cancer, Non-steroidal anti-inflammatory drug, Hormone receptor positive breast cancer, HER2 positive breast cancer, Triple negative breast cancer

## Abstract

**Background:**

The relationship between non-steroidal anti-inflammatory drug (NSAID) consumption and breast cancer has been repeatedly studied, although the results remain controversial. Most case-control studies reported that NSAID consumption protected against breast cancer, while most cohort studies did not find this effect. Most studies have dealt with NSAIDs as a whole group or with specific drugs, such aspirin, ibuprofen, or others, but not with NSAID subgroups according to the Anatomical Therapeutic Chemical Classification System; moreover, scarce attention has been paid to their effect on different tumor categories (i.e.: ductal/non-ductal, stage at diagnosis or presence of hormonal receptors).

**Methods:**

In this case-control study, we report the NSAID – breast cancer relationship in 1736 breast cancer cases and 1895 healthy controls; results are reported stratifying by the women’s characteristics (i.e.: menopausal status or body mass index category) and by tumor characteristics.

**Results:**

In our study, NSAID use was associated with a 24 % reduction in breast cancer risk (Odds ratio [OR] = 0.76; 95 % Confidence Interval [CI]: 0.64–0.89), and similar results were found for acetic acid derivatives, propionic acid derivatives and COXIBs, but not for aspirin. Similar results were found in postmenopausal and premenopausal women. NSAID consumption also protected against hormone + or HER2+ cancers, but not against triple negative breast cancers. The COX-2 selectivity showed an inverse association with breast cancer (i.e. OR < 1), except in advanced clinical stage and triple negative cancers.

**Conclusion:**

Most NSAIDs, but not aspirin, showed an inverse association against breast cancer; this effect seems to be restricted to hormone + or HER2+ cancers.

**Electronic supplementary material:**

The online version of this article (doi:10.1186/s12885-016-2692-4) contains supplementary material, which is available to authorized users.

## Background

The cyclooxygenase-prostaglandin inflammation pathway has been shown to play a relevant role in carcinogenesis, mainly via inhibition of the cyclooxigenase-2 (COX-2) isoform [1]. Experimental studies have demonstrated that COX-2 blockade inhibits breast tumor formation in mice, while its overexpression has the opposite effect [2]. Therefore, consumption of non-steroidal anti-inflammatory drugs (NSAIDs) is expected to be protective for cancer development.

Regarding breast cancer, results from epidemiological studies are inconsistent: cohort studies have reported very modest protective effects or no effect at all [3–5], while case-control studies have usually reported moderate protective effects [6–8]. Several meta-analyses have been conducted; combination of results is, however, complex because of differences in reporting of timing and dosing of NSAIDs in the studies. The most recent meta-analysis reported a 20 % protective effect of NSAID especially aspirin and COX-2 inhibitors against breast cancer, which seems to be restricted to estrogen receptors + (ER+) or progesterone receptors + (PR+) tumors [9].

The number of epidemiological studies reporting results about the COX-2 inhibitors’ effect on breast cancer or about NSAIDs’ effect on different types of breast cancer (i.e.: ER+, PR+, Human epidermal growth factor receptor [HER2] +, triple negative) is still small and further studies are needed in order to clarify the specific effect of NSAID groups on different types of breast cancer [8, 10–12]. In order to further investigate this issue, we report the results from a large case-control study performed in Spain.

## Methods

### Study design and population

The Multi Case-control (MCC-Spain) study is a population-based case-control study of common tumors in Spain and has been described elsewhere [13]. It has been carried out in 12 Spanish provinces. The recruitment included incident cases of colorectal, breast, stomach and prostate cancer or chronic lymphocytic leukemia diagnosed between September 1st, 2008 and December 31st, 2013, aged between 20 and 85 years old, and resident within the influence area of the hospital at least 6 months prior to recruitment. Cases were identified through active search that included periodical visits to the collaborating hospital departments (i.e. gynecology, oncology, general surgery, radiotherapy, and pathology departments), but only histologically confirmed incident cases of breast cancer (C50, D05.1, D05.7) with no prior history of the disease were included in this study. Ten out of 12 provinces recruited breast cancer cases and controls. Controls were selected from the general population according to age and sex distribution of the cases included in the study. In this paper, 1736 cases of breast cancer (ICD-10: C50, D05) in women and 1909 frequency-matched controls were considered.

Response rates were 71 % for breast cancer and 72 % for controls, with no differences in the main socio-demographic variables among those who participated and those who refused to participate. The Ethics Committees of participating hospitals approved the study protocols, and participants provided written informed consent at the time of enrollment.

### Data collection

Participants were interviewed face-to-face by trained interviewers with a comprehensive epidemiological questionnaire that assessed socio-demographic information, personal and family history of cancer, anthropometric data, smoking habits, occupation, physical activity, water consumption, reproductive and medical history and medication/drugs use, family history, sun exposure, sleep habits, use of hygiene products and cosmetics, signs and symptoms. Diet was assessed with the use of a validated semi-quantitative Spanish Food Frequency Questionnaire (FFQ), which was modified to include regional products. The FFQ included 140 food items, and assessed usual dietary intake during the previous year.

Participant’s weight was recorded by self-report, as estimated one year before diagnosis for cases and for controls. Body mass index (BMI) was estimated from self-reported weight and height 1 year before the diagnosis for cases and 1 year prior to the interview for controls. Similar estimates provided total energy consumption. Physical activity was recorded for all jobs and also recreational physical exercise.

Detailed information was obtained on past medical conditions and the corresponding medications used. Participants were asked about past medical history of diabetes mellitus, high blood pressure, high levels of cholesterol and triglycerides, heart attack, embolism, other cardiovascular diseases, degenerative osteoarthritis, arthritis, migraine or cephalalgia, gout, ulcerative colitis, Crohn’s disease, renal calculus (nephrolithiasis or cystolithiasis), chronic obstructive pulmonary disease, asthma, bronchitis, irritable bowel syndrome, anemia, diverticulitis, celiac disease and cancer. The age at onset, the dates of diagnosis or occurrence and the type of treatment received for each condition was also registered.

### Drug use assessment

Drug use was recorded by indication. For each drug, the brand name, dose and duration of exposure were recorded to identify patients with regular drug consumption (“no and occasionally” versus “yes”) and the duration of consumption. The drugs were coded following the Anatomical Therapeutic Chemical Classification System (ATC codes) to define groups with similar mechanisms of action [14]. To be sure that participants report all drugs, a general question about the use of NSAIDs was included in order to add information that was not provided before.

All drugs indicated for the treatment of inflammatory diseases were considered. The main ATC code included in the present analysis are codes B01AC06 and N02BA01 (Aspirin) and code M01 (Antiinflammatory and antirheumatic drugs). Data were also analyzed for subgroups with codes M01AA (Butilpirazolidins), M01AB (acetic acid derivatives; for instance, diclofenac, ketorolac), M01AC (Oxicams), M01AE (propionic acid derivatives; for instance ibuprofen, naproxen), M01AH (Coxibs; for instance, celecoxib), M01AX (other NSAIDs) and their combinations. Finally, as cox2 inhibition has been suggested as the putative mechanisms for NSAID protective effect on breast cancer, we performed a subgroup analysis according to level of COX-selectivity. In this way, NSAIDs were grouped in cox1-selective/cox2-selective according to their log [IC80 ratio (WHMA COX-2/COX-1)] [15]. NSAIDs with negative log (IC80 ratio) were considered cox2-selective (for instance, meloxicam, diclofenac, sulindac, piroxicam, niflumic acid), while NSAIDs with positive log (IC80 ratio) were considered cox1-selective (for instance: ibuprofen, naproxen, indomethacin, ketoprofen, ketorolac). As the putative protective mechanism of aspirin is not via cox-2 inhibition, we retained aspirin as an independent group.

### Statistical methods

Unconditional logistic regression was used to assess the association between treatment of NSAID use and breast cancer, adjusting for age, recruitment area, education level, tobacco smoking history, BMI, family history of breast cancer, number of deliveries, age at first delivery, menarche age, and menopausal status. Stratified models were developed according to menopausal status and BMI [<25/≥25 kg/m^2^]. The association between tumor characteristics (clinical stage, ductal/non-ductal cancer, hormone receptors, HER2 receptors and triple negative breast cancer) and NSAID consumption was studied using multinomial logistic regression. Results are reported as odds ratios (OR) with 95 % confidence intervals (CI). All reported *p*-values are two-tailed. Statistical analysis was carried out using the package Stata 12/SE (StataCorp, College Station, Tx, US).

## Results

A description of the 1736 cases and 1909 controls included in this study is provided in Table 1. Significant differences are observed between cases and controls for several well known risk factors for breast cancer, including family history of breast cancer, age at menarche, and tobacco smoking. Clinical-pathological characteristics of the breast cancers are reported in Table 2; ductal cancer accounts for 85 % of cases; two out of three breast cancers were diagnosed at stage I or II; more than 70 % of cancers were estrogen receptors +, 14 % were HER2 receptors + and only 6 % were triple negative breast cancers. Results on NSAID consumption – breast cancer association are reported here for consumption of any NSAID, aspirin, acetic acid derivatives, propionic acid derivatives, COX-2 inhibitors (COXIBs), and other NSAIDs. We do not report results on butilpirazolidins because of the small number of women exposed to this group.

**Table 1:** Main characteristics of cases and controls from the study population

Variable	Category	Cases	Controls	p
Age, mean ± sd		56.4 ± 12.6	59.0 ± 13.2	<0.001
Geographical area, n (%)	Asturias	70 (4.0)	121 (6.4)	
	Barcelona	292 (16.8)	380 (20.1)	
	Cantabria	141 (8.1)	188 (9.9)	
	Gerona	47 (2.7)	57 (3.0)	
	Guipuzcoa	226 (13.0)	255 (13.5)	
	Huelva	105 (6.1)	79 (4.2)	
	Leon	227 (13.1)	202 (10.7)	
	Madrid	341 (19.6)	365 (19.3)	
	Navarra	226 (13.0)	181 (9.6)	
	Valencia	61 (3.5)	67 (3.5)	<0.001
Family history of breast cancer, n (%)	No	1288 (75.0)	1628 (85.7)	
	First-degree relative	256 (14.9)	166 (8.7)	
	Second-degree relative	174 (10.1)	106 (5.8)	<0.001
Educational level, n (%)	Less than primary school	268 (15.4)	327 (17.3)	
	Primary school	565 (32.6)	581 (30.7)	
	Secondary school	573 (33.0)	585 (30.9)	
	University	330 (19.0)	402 (21.2)	0.10
Tobacco smoking, n (%)	Never smoker	972 (56.0)	1141 (60.2)	
	Former smoker	450 (25.9)	397 (21.0)	
	Current smoker	314 (18.1)	357 (18.8)	0.002
Body Mass Index (kg/m^2^), n (%)	<18.5	30 (1.7)	43 (2.3)	
	18.5–24.9	789 (45.5)	899 (47.4)	
	25.0–29.9	590 (34.0)	601 (31.7)	
	≥30	327 (18.8)	352 (18.6)	0.31
Energy intake (kcal/day), mean ± sd		1861 ± 644	1754 ± 566	<0.001
Ethanol intake (g/day), mean ± sd		6.2 ± 11.5	5.3 ± 9.5	0.01
Red meat intake (g/day), mean ± sd		26.9 ± 20.2	25.2 ± 19.9	0.01
Fruit intake (g/day), mean ± sd		363 ± 239	365 ± 222	0.87
Vegetable intake (g/day), mean ± sd		196 ± 133	198 ± 119	0.60
Dairy intake (g/day), mean ± sd		321.1 ± 177.1	328.7 ± 176.7	0.24
Number of deliveries, mean ± sd		1.9 ± 1.5	2.0 ± 1.6	0.03
Menopausal status, n (%)	Premenopausal	702 (40.4)	628 (33.1)	
	Postmenopausal	1034 (59.6)	1267 (66.9)	<0.001
Age at first delivery, mean ± sd		26.5 ± 5.0	26.5 ± 4.7	0.82
Age at menarche, mean ± sd		12.8 ± 1.5	12.9 ± 1.5	0.02
Age at menopause, mean ± sd		48.8 ± 5.4	48.5 ± 5.3	0.18
Previous use of hormonal contraceptives, n (%)		789 (45.5)	868 (45.8)	0.83

**Table 2:** Clinical and pathological characteristics of breast cancers

Classification	N (%)
Pathology	
Ductal	1289 (74.3)
Lobular	112 (6.5)
Papilar	22 (1.3)
Coloid	20 (1.2)
Tubular	12 (0.7)
Mixed	27 (1.6)
Other	35 (2.0)
Not Available	213 (12.3)
Clinical stage	
0	115 (6.6)
I	604 (34.8)
II	495 (28.5)
III	182 (10.5)
IV	22 (1.3)
Not Available	196 (11.3)
Inmunohistochemistry	
Progesterone	992 (62.5)
Estrogens	1147 (72.2)
HER2	227 (14.3)
Triple negative	92 (5.8)

### NSAID consumption and breast cancer according to women’s characteristics

Results on the relationship between NSAID consumption and breast cancer overall and by menopausal status and BMI, according to women’s characteristics are reported in Tables 3 and 4 for duration of use, and Additional file 1: Table S1 according to COX2/COX1 selectivity. NSAIDs as a global group protected against breast cancer (OR = 0.76; 95 % CI: 0.64–0.89); a protective effect was also found for acetic acid derivatives, propionic acid derivatives and COXIBs, but not for aspirin, although COXIB results were based on small numbers of exposed cases and controls, hampering further analysis of their effect in specific subgroups of women. When stratifying for menopausal status, all NSAIDs, acetic acid derivatives, propionic acid derivatives and COXIBs showed a protective effect in postmenopausal women; ORs in postmenopausal women were similar or slightly lower to those in premenopausal women. P values for NSAID – menopausal interaction status were higher than 0.10 (*p* values not shown). The protective effect of any NSAID was independent of BMI; however, the effect varied in subgroups: acetic acid derivatives were protective in women with BMI < 25 kg/m^2^ (OR = 0.54; 95 % CI: 0.31–0.93) but not in overweight or obese women, while propionic acid derivatives (OR = 0.78; 95 % CI: 0.61–1.00) protected only in the latter group; p values for BMI – NSAID interaction were non-significant. Table 4 reports the results according to the duration of NSAID consumption (never/less than 5 years/more than 5 years). It shows that most of the results described in the paragraph above had consistent dose-effect relationship: the longer the consumption, the lower the odds ratio. Additional file 1: Table S1 shows a greater protective effect of COX-2 both globally (OR = 0.66; 95 % CI: 0.48–0.90 for COX-2 vs OR = 0.81; 95 % CI: 0.67–0.98) for COX-1 selectivity) and in postmenopausal women and in women with BMI <25 kg/m^2^.

**Table 3:** Relationship between NSAID consumption and breast cancer according to women’s characteristics

Population	NSAID	Unexposed controls/cases (n)	Exposed controls/Cases (n)	OR (95 % CI)	p
All women	NSAID (all)	1170/1111	739/625	0.76 (0.64–0.89)	0.001
	Aspirin	1807/1653	102/83	0.91 (0.64–1.29)	0.60
	Acetic acid derivatives	1753/1620	156/116	0.75 (0.55–1.01)	0.06
	Propionic acid derivatives	1350/1232	559/504	0.82 (0.69–0.98)	0.03
	cox2 inhibitors	1891/1731	18/5	0.28 (0.09–0.88)	0.03
	NSAID others	1861/1697	48/39	1.13 (0.67–1.89)	0.65
Premenopausal	NSAID (all)	361/415	267/287	0.80 (0.60–1.07)	0.14
	Aspirin	613/689	15/13	0.60 (0.25–1.47)	0.27
	Acetic acid derivatives	600/664	28/38	0.75 (0.42–1.36)	0.35
	Propionic acid derivatives	392/451	236/251	0.84 (0.62–1.13)	0.24
	cox2 inhibitors	626/701	2/1	-	-
	NSAID others	618/693	10/9	1.26 (0.41–3.9)	0.69
Postmenopausal	NSAID (all)	804/696	462/338	0.69 (0.56–0.86)	<0.001
	Aspirin	1180/964	86/70	0.99 (0.68–1.46)	0.98
	Acetic acid derivatives	1142/956	124/78	0.72 (0.50–1.03)	0.07
	Propionic acid derivatives	953/781	313/253	0.78 (0.62–0.98)	0.03
	cox2 inhibitors	1250/1030	16/4	-	-
	NSAID others	1228/1004	38/30	1.05 (0.58–1.90)	0.86
BMI <25	NSAID (all)	578/537	364/282	0.73 (0.57–0.94)	0.02
	Aspirin	901/794	41/25	0.74 (0.40–1.35)	0.32
	Acetic acid derivatives	883/783	59/36	0.54 (0.31–0.93)	0.03
	Propionic acid derivatives	656/575	286/244	0.86 (0.66–1.13)	0.27
	cox2 inhibitors	932/816	10/3	-	-
	NSAID others	919/806	23/13	0.98 (0.42–2.31)	0.97
BMI >25	NSAID (all)	592/574	375/343	0.76 (0.60–0.95)	0.02
	Aspirin	906/859	61/58	1.01 (0.65–1.56)	0.96
	Acetic acid derivatives	870/837	97/80	0.81 (0.56–1.18)	0.27
	Propionic acid derivatives	694/657	273/260	0.78 (0.61–1.00)	0.05
	cox2 inhibitors	959/915	8/2	-	-
	NSAID others	942/891	25/26	1.24 (0.64–2.41)	0.52

*OR* Odds ratio adjusted for age, recruitment area, education level, tobacco smoking history, *BMI* family history of breast cancer, number of deliveries, age at first delivery, menarche age, and menopausal status. *CI* confidence interval

**Table 4:** Relationship between length of non-steroideal anti-inflammatory drug consumption and breast cancer, according to women’s characteristics

Population	NSAID	No consumption	Consumption ≤5y		Consumption >5y	
		Controls/cases (n)	Controls/cases (n)	OR (95 % CI)	Controls/cases (n)	OR (95 % CI)
All women	NSAID (all)	1171/1111	484/445	0.81 (0.67–0.98)	255/180	0.64 (0.50–0.83)
	Aspirin	1808/1653	79/70	0.99 (0.67–1.46)	23/13	0.65 (0.31–1.38)
	Acetic acid derivatives	1754/1620	113/86	0.75 (0.53–1.06)	43/30	0.75 (0.42–1.32)
	Propionic acid derivatives	1351/1232	380/364	0.86 (0.71–1.06)	179/140	0.73 (0.55–0.97)
	NSAID others	1862/1697	33/35	1.47 (0.83–2.60)	15/4	-
Premenopausal	NSAID (all)	361/415	162/187	0.82 (0.59–1.13)	105/100	0.78 (0.52–1.17)
	Aspirin	613/689	12/12	0.72 (0.27–1.95)	3/1	-
	Acetic acid derivatives	600/664	21/27	0.68 (0.35–1.34)	7/11	1.02 (0.31–3.32)
	Propionic acid derivatives	392/461	146/167	0.85 (0.61–1.19)	90/84	0.81 (0.52–1.25)
	NSAID others	618/693	6/8	2.38 (0.63–9.00)	4/1	-
Postmenopausal	NSAID (all)	805/696	312/258	0.77 (0.61–0.98)	150/80	0.53 (0.38–0.75)
	Aspirin	1181/964	66/58	1.08 (0.70–1.66)	20/12	0.74 (0.33–1.65)
	Acetic acid derivatives	1143/956	88/59	0.76 (0.50–1.15)	36/19	0.61 (0.31–1.20)
	Propionic acid derivatives	954/781	224/197	0.84 (0.64–1.09)	89/56	0.64 (0.43–0.95)
	NSAID others	1229/1004	27/27	1.27 (0.66–2.42)	11/3	-
BMI <25	NSAID (all)	578/537	233/183	0.78 (0.58–1.04)	131/99	0.65 (0.45–0.94)
	Aspirin	901/794	32/22	0.84 (0.43–1.63)	9/3	-
	Acetic acid derivatives	883/783	44/22	0.40 (0.20–0.77)	15/14	1.16 (0.42–3.15)
	Propionic acid derivatives	656/575	191/163	0.89 (0.66–1.21)	95/81	0.80 (0.52–1.22)
	NSAID others	919/806	14/11	1.51 (0.56–4.06)	9/2	-
BMI >25	NSAID (all)	593/574	251/262	0.84 (0.65–1.08)	124/81	0.61 (0.43–0.86)
	Aspirin	907/859	47/48	1.10 (0.68–1.80)	14/10	0.74 (0.30–1.82)
	Acetic acid derivatives	871/837	69/64	0.94 (0.61–1.44)	28/16	0.54 (0.27–1.10)
	Propionic acid derivatives	695/657	189/201	0.85 (0.64–1.12)	84/59	0.64 (0.43–0.95)
	NSAID others	943/891	19/24	1.44 (0.71–2.95)	6/2	-

*OR* Odds ratio adjusted for age, recruitment area, education level, tobacco smoking history, *BMI* family history of breast cancer, number of deliveries, age at first delivery, menarche age, and menopausal status. *CI* confidence interval

### NSAID consumption and breast cancer according to tumor characteristics

Results for subgroups of breast cancer are reported in Tables 5 and 6 (for duration of use) and Additional file 2: Table S2 (according to COX2/COX1 selectivity). The protective effect of any NSAID seemed similar in early or late clinical stages (OR = 0.80; 95 % CI: 0.66–0.97 in stages 1–2; OR = 0.74; 95 % CI: 0.51–1.06 in stages 3–4), but no specific NSAID group reached statistically significant effect. Consumption of any NSAID, acetic acid derivatives and propionic acid derivatives was protective for ductal cancer (OR for any NSAID = 0.70; 95 % CI: 0.58–0.84) but not for non-ductal cancer, although the p value for heterogeneity was non-significant for NSAID as a group or for any specific subgroup. All NSAID consumption was protective for hormone receptor + cancer (i.e.: ER+ or PR+) (OR = 0.72; 95 % CI: 0.60–0.88) and HER2+ cancers (OR = 0.63; 95 %: 0.45–0.88). Propionic acid derivatives also showed this protective effect in hormone + or HER2+ cancers, while acetic acid derivatives showed a non-statistically significant effect (OR = 0.76; 95 % CI: 0.54–1.08 in hormone receptor + cancers and OR = 0.67; 95 % CI: 0.36–1.24 in HER2 receptor + cancers). Neither consumption of NSAID in general nor any specific NSAID subgroup showed a protective effect in triple negative breast cancers.

**Table 5:** Relationship between consumption of non-steroideal anti-inflammatory drugs and breast cancer, according to tumor characteristics

Variable	Category	NSAID	Unexposed controls/cases (n)	Exposed controls/cases (n)	OR (95 % CI)	P
Clinical stage	1–2	NSAID (all)	1170/696	739/404	0.80 (0.66–0.97	0.02
		Aspirin	1807/1047	102/52	0.93 (0.63–1.38)	0.72
		Acetic acid derivatives	1753/1025	156/74	0.75 (0.54–1.06)	0.11
		Propionic acid derivatives	1350/770	559/329	0.90 (0.74–1.11)	0.33
		cox2 inhibitors	1891/1095	18/4	-	-
		NSAID others	1861/1072	48/27	1.07 (0.61–1.90)	0.81
	3–4	NSAID (all)	1170/136	739/68	0.74 (0.51–1.06)	0.10
		Aspirin	1807/192	102/12	1.31 (0.66–2.59)	0.44
		Acetic acid derivatives	1753/190	156/14	0.99 (0.53–1.83)	0.97
		Propionic acid derivatives	1350/147	559/57	0.84 (0.57–1.24)	0.39
		cox2 inhibitors	1891/204	18/0	-	-
		NSAID others	1861/201	48/3	-	-
Pathology	Ductal cancer	NSAID (all)	1170/835	739/454	0.70 (0.58–0.84)	<0.001
		Aspirin	1807/1225	102/64	0.97 (0.66–1.41)	0.86
		Acetic acid derivatives	1753/1204	156/85	0.76 (0.55–1.06)	0.11
		Propionic acid derivatives	1350/918	559/371	0.78 (0.64–0.95)	0.01
		cox2 inhibitors	1891/1289	18/3	-	-
		NSAID others	1861/1259	48/30	1.06 (0.60–1.87)	0.85
	Non-ductal cancer	NSAID (all)	1170/151	739/83	0.82 (0.58–1.15)	0.25
		Aspirin	1807/228	102/6	0.50 (0.21–1.19)	0.12
		Acetic acid derivatives	1753/219	156/15	0.85 (0.46–1.58)	0.61
		Propionic acid derivatives	1350/166	559/68	0.91 (0.63–1.31)	0.60
		cox2 inhibitors	1891/234	18/0	-	-
		NSAID others	1861/232	48/2	-	-
Inmunohistochemistry	Hormone +	NSAID (all)	1170/727	739/390	0.72 (0.60–0.88)	<0.001
		Aspirin	1807/1069	102/48	0.82 (0.55–1.24)	0.35
		Acetic acid derivatives	1753/1044	156/73	0.76 (0.54–1.08)	0.12
		Propionic acid derivatives	1350/805	559/312	0.80 (0.65–0.98)	0.03
		cox2 inhibitors	1891/1115	18/2	-	-
		NSAID others	1861/1089	48/28	1.28 (0.73–2.25)	0.38
	HER2+	NSAID (all)	1170/739	172/83	0.63 (0.45–0.88)	0.007
		Aspirin	1807/102	244/11	0.79 (0.38–1.65)	0.53
		Acetic acid derivatives	1753/238	156/17	0.67 (0.36–1.24)	0.20
		Propionic acid derivatives	1350/188	559/67	0.66 (0.46–0.95)	0.03
		cox2 inhibitors	1891/255	18/0	-	-
		NSAID others	1861/251	48/4	-	-
	Triple negative breast cancer	NSAID (all)	1170/94	739/63	0.87 (0.58–1.30)	0.49
		Aspirin	1807/148	102/9	1.24 (0.57–2.71)	0.59
		Acetic acid derivatives	1753/147	156/10	0.86 (0.41–1.79)	0.68
		Propionic acid derivatives	1350/103	559/54	0.99 (0.64–1.52)	0.95
		cox2 inhibitors	1891/156	18/1	-	-
		NSAID others	1861/154	48/3	-	-

*OR* Odds ratio adjusted for age, recruitment area, education level, tobacco smoking history, *BMI* family history of breast cancer, number of deliveries, age at first delivery, menarche age, and menopausal status. *CI* confidence interval

**Table 6:** Relationship between length of non-steroideal anti-inflammatory drug consumption and breast cancer, according to tumor characteristics

Variable	Category	NSAID	No consumption	Consumption ≤5y		Consumption >5y	
			Controls/cases (n)	Controls/cases (n)	OR (95 % CI)	Controls/cases (n)	OR (95 % CI)
Clinical stage	1–2	NSAID (all)	1171/695	484/281	0.85 (0.69–1.06)	255/123	0.69 (0.52–0.92)
		Aspirin	1808/1047	79/44	1.01 (0.65–1.58)	23/8	0.74 (0.31–1.73)
		Acetic acid derivatives	1754/1025	113/53	0.79 (0.53–1.17)	43/21	0.73 (0.38–1.42)
		Propionic acid derivatives	1351/770	380/234	0.93 (0.73–1.17)	179/95	0.78 (0.57–1.08)
		NSAID others	1862/1072	33/24	1.56 (0.82–2.95)	15/3	-
	3–4	NSAID (all)	1171/136	484/54	0.85 (0.57–1.27)	255/14	0.46 (0.24–0.88)
		Aspirin	1808/192	79/11	1.61 (0.78–3.34)	23/1	-
		Acetic acid derivatives	1754/190	113/12	1.08 (0.54–2.17)	43/2	-
		Propionic acid derivatives	1351/147	380/45	0.91 (0.59–1.40)	179/12	0.54 (0.27–1.09)
		NSAID others	1862/201	33/3	-	15/0	-
Pathology	Ductal cancer	NSAID (all)	1171/835	484/324	0.74 (0.60–0.91)	255/130	0.60 (0.45–0.79)
		Aspirin	1808/1225	79/56	1.09 (0.72–1.66)	23/8	0.60 (0.26–1.41)
		Acetic acid derivatives	1754/1204	113/64	0.78 (0.53–1.14)	43/21	0.68 (0.36–1.30)
		Propionic acid derivatives	1351/918	380/270	0.81 (0.65–1.02)	179/101	0.68 (0.49–0.94)
		NSAID others	1862/1259	33/28	1.42 (0.77–2.67)	15/2	
	Non-ductal cancer	NSAID (all)	1171/151	484/50	0.78 (0.53–1.15)	255/33	0.90 (0.55–1.48)
		Aspirin	1808/228	79/5	0.59 (0.23–1.53)	23/1	-
		Acetic acid derivatives	1754/219	113/11	0.92 (0.45–1.87)	43/4	-
		Propionic acid derivatives	1351/166	380/40	0.79 (0.51–1.22)	179/28	1.15 (0.67–1.96)
		NSAID others	1862/232	33/1	-	15/1	-
Inmunohisto-chemistry	Hormone +	NSAID (all)	1171/727	484/267	0.74 (0.60–0.92)	256/123	0.69 (0.52–0.92)
		Aspirin	1808/1069	79/38	0.85 (0.54–1.36)	23/10	0.79 (0.36–1.75)
		Acetic acid derivatives	1754/1044	113/50	0.69 (0.46–1.04)	43/23	0.94 (0.51–1.75)
		Propionic acid derivatives	1351/805	380/221	0.82 (0.65–1.04)	179/91	0.76 (0.55–1.05)
		NSAID others	1862/1089	33/25	1.62 (0.87–3.01)	15/3	-
	HER2 +/Hormone-	NSAID (all)	1171/172	484/61	0.70 (0.48–1.01)	255/22	0.49 (0.28–0.87)
		Aspirin	1808/244	79/10	0.89 (0.41–1.96)	23/1	-
		Acetic acid derivatives	1754/238	113/16	1.02 (0.29–3.60)	43/1	-
		Propionic acid derivatives	1351/188	380/47	0.66 (0.43–0.99)	179/20	0.61 (0.33–1.12)
		NSAID others	1862/251	33/4	-	15/0	-
	Triple negative breast cancer	NSAID (all)	1171/94	484/49	0.94 (0.60–1.48)	255/14	0.60 (0.30–1.17)
		Aspirin	1808/148	79/9	1.74 (0.78–3.89)	23/0	-
		Acetic acid derivatives	1754/144	113/8	0.85 (0.36–2.01)	43/2	-
		Propionic acid derivatives	1351/103	380/42	1.04 (0.64–1.68)	179/12	0.68 (0.32–1.43)
		NSAID others	1862/154	33/2	-	15/1	-

*OR* odds ratio adjusted for age, recruitment area, education level, tobacco smoking history, *BMI* family history of breast cancer, number of deliveries, age at first delivery, menarche age, and menopausal status. *CI*: confidence interval

When studying the effect of length of consumption (Table 6), most associations reported above were at least as strong in patients with more than 5 years of consumption as in patients with less than 5 years.

Finally, regarding the COX-selectivity of the NSAID (Additional file 2: Table S2), the COX-2 selectivity showed an inverse association with breast cancer (i.e. OR < 1), except in advanced clinical stage and triple negative cancers.

## Discussion

In this large case-control study, NSAID use was associated with a 24 % reduction in breast cancer risk. An inverse association were observed specifically for acetic acid derivative and propionic acid derivative use, but not for aspirin consumption. There is a trend towards a stronger protective effect of NSAID in postmenopausal women, ductal cancer, and hormone receptor or HER2 receptor positive tumors. This protective effect was less pronounced in premenopausal women, non-ductal cancer, or triple negative cancer, although the small number of cases with triple negative cancer makes it difficult to reach definitive conclusions.

Regarding NSAID effect overall, our results are coherent with those reported in 10 out of 16 case-control studies [6–8, 16–22], while the remaining six studies did not show any effect [23–28]. Results from 13 cohort studies hardly support any NSAID effect on breast cancer risk; only four reported protective effects [3, 4, 29, 30], seven did not find any association [5, 31–35], and three reported an increase in breast cancer risk [36–38]. Consequently, a recent meta-analysis [9] showed a significant protective odds ratio (OR = 0.82) when combining case-control studies, but a non-significant relative risk (RR = 0.92) in cohort studies. Most studies, however, did not report stratified results.

Some studies have analyzed the effect of aspirin, ibuprofen or non-aspirin NSAIDs, reporting similar results to those presented for NSAIDs in general (i.e.: protective effect in case-control studies; no effect in cohort studies) [9]. Scarce attention has been paid, however, to the effect associated with different pharmacological subgroups. According to our results, acetic acid derivatives, propionic acid derivatives and COX-2 inhibitors have a protective effect against breast cancer incidence, while aspirin has no effect at all. The absence of a significant effect of aspirin is puzzling since prior investigations have noted not only preventive effects but also therapeutic effects of aspirin against breast cancer. In this regard, aspirin consumption could be underreported in our study due to its common over-the-counter usage; as this possible underreporting would affect both cases and controls in a similar way, it would eventually lead to a bias towards the null, which would justify a negative result. This phenomenon is not to be expected in other NSAIDs as their usual consumption is by prescription.

The public health implications of the reduction in breast cancer risk when taking acetic acid and propionic acid derivatives should be highlighted as these groups account for about 80 % of NSAID consumption in the Spanish population. A note of caution should be remarked on aspirin results; the percentage of people declaring aspirin consumption seems low, which could be due to a reporting bias. Study participants were asked to report the diseases they were suffering from and the drugs they had been taking for treating them and we have also asked whether they were taking any other NSAID not reported before. It is possible that some people might not consider aspirin to be a drug, so failing to declare its usage.

Most studies did not analyze NSAID effect on several types of breast cancer. According to our results, the inverse association of NSAID with breast cancer is more pronounced in postmenopausal cancers, ductal cancer, and hormone receptor or HER2 receptor positive tumors. This effect increases in women treated with COX-2 inhibitors, especially in early clinical stage, postmenopausal cancers and receptor positive tumors. The putative pathway for the NSAID protective effect is via COX inhibition. High levels of prostaglandins, derived from the activation of the COX/prostaglandin pathway, contribute to carcinogenesis in various ways (increase in mitogenesis, mutagenesis, angiogenesis, metastasis formation, inhibition of apoptosis, and immunosuppression) [38–40]. Constitutive expression of the COX-2 gene and sustained biosynthesis of PGE_2_ seem to be associated with the initiation and promotion of breast carcinogenesis [41]. In a prospective study, COX-2 expression in biopsy specimens from women with atypical breast hyperplasia was a significant predictor of breast cancer risk [42]; COX-2 overexpression, therefore, seems to constitute an early event in breast carcinogenesis, which makes COX-2 a potential cancer biomarker and a key target for breast cancer prevention [43]. Unfortunately, cardiovascular toxicity attributed to COX-2 inhibitors has partially decreased their usefulness, whatever their effect on breast cancer might be.

On the other hand, COX inhibition would reduce aromatase activity [44]. Peripheral aromatization of fatty acids is known to be largely responsible for estrogen production in postmenopausal women –in whom adipose tissue represents an important local source of estrogen-; therefore, regulation of aromatase synthesis in the breast could be particularly important in postmenopausal breast cancer [45]. Reducing aromatase activity via COX inhibition could also explain, at least partially, the decrease in breast cancer incidence linked to NSAID use [8], since COX inhibition would reduce estrogen concentration in the breast, restricting the growth of estrogen-dependent tumors.

This study has several limitations. First, NSAID consumption was self-reported, which could introduce a recall bias. For a recall bias to be responsible for the protective effects reported here, the bias would have to be differential in cases and controls, with controls remembering their previous NSAID consumption better; this seems counterintuitive as one would expect cases to be more motivated for remembering their previous exposures. In addition, if cases are less prone to report their NSAID usage, the same bias would be expected in all NSAID groups and in each stratum analyzed; however, our results were different according to the type of NSAID, which seems to contradict such a bias. Moreover, in order to minimize a differential recall bias, interviewers were blinded to the case-control status of the participants. Second, although our intention was to record data on aspirin dosage, most patients did not provide sufficiently detailed data on dosages of aspirin or other NSAID use. This fact prevents us from analyzing the dose-effect relationship. Third, we have adjusted for the usual confounders but residual confounding cannot be ruled out. Finally, any case-control study could be affected by a selection bias. Our study is population based, as controls have been selected from the same residence area as cases; the small differences in case and control educational levels suggest that the selection has been adequately carried out. Moreover, the high response rates obtained in this study (71 % for breast cancer cases and 72 % for controls, respectively) minimize the possibility of occurrence of such bias.

## Conclusions

Summarizing, although there is increasing evidence for a protective effect of NSAID against breast cancer risk, our results indicate that this effect is more pronounced in postmenopausal women and in estrogen/progesterone + receptor or HER2+ cancers. As this effect seems to be moderate, concerns remain about whether NSAID may play a role in chemoprevention or just indicate a pathway for identifying further more specific drugs that could be used for breast cancer chemoprevention in high risk women.

## Additional files

Additional file 1: Table S1. Relationship between NSAID consumption and breast cancer according to COX2/COX1 selectivity and women’s characteristics (DOC 34 kb) Additional file 2: Table S2. Relationship between NSAID consumption and breast cancer according to COX2/COX1 selectivity and tumor characteristics (DOC 39 kb)
