# Supplementary material for: Use of non-steroidal anti-inflammatory drugs and risk of breast cancer: The Spanish Multi-Case-control (MCC) study
Source: BMC Cancer. 2016 Aug 20;16:660. doi: 10.1186/s12885-016-2692-4 (PMC4992258; doi:10.1186/s12885-016-2692-4)
Supplement: Additional file 1: Table S1. — Relationship between NSAID consumption and breast cancer according to COX2/COX1 selectivity and women’s characteristics (DOC 34 kb) [file 12885_2016_2692_MOESM1_ESM.doc]

Additional file 1: Table S1. **Relationship between non-aspirin NSAID consumption and breast cancer according to COX2/COX1 selectivity and women’s characteristics**

| **Population** | **NSAID** | **OR (95% CI)** | **p** |
| --- | --- | --- | --- |
| **All women** | cox1 selective | 0.81 (0.67-0.98) | 0.0270 |
|  | cox2 selective | 0.66 (0.48- 0.90) | 0.0095 |
| **Premenopausal** | cox1 selective | 0.84 (0.62-1.15) | 0.2750 |
|  | cox2 selective | 0.90 (0.48- 1.69) | 0.7362 |
| **Postmenopausal** | cox1 selective | 0.75 (0.59-0.95) | 0.0194 |
|  | cox2 selective | 0.53 (0.36-0.77) | 0.0010 |
| **BMI<25** | cox1 selective | 0.82 (0.62-1.08) | 0.1565 |
|  | cox2 selective | 0.48 (0.27- 0.85) | 0.0114 |
| **BMI>25** | cox1 selective | 0.79 (0.61- 1.02) | 0.0661 |
|  | cox2 selective | 0.73 (0.49-1.09) | 0.1196 |

OR: Odds ratio adjusted for age, recruitment area, education level, tobacco smoking history, BMI, family history of breast cancer, number of deliveries, age at first delivery, menarche age, and menopausal status. CI: confidence interval
