# Supplementary material for: Use of non-steroidal anti-inflammatory drugs and risk of breast cancer: The Spanish Multi-Case-control (MCC) study
Source: BMC Cancer. 2016 Aug 20;16:660. doi: 10.1186/s12885-016-2692-4 (PMC4992258; doi:10.1186/s12885-016-2692-4)
Supplement: Additional file 2: Table S2. — Relationship between NSAID consumption and breast cancer according to COX2/COX1 selectivity and tumor characteristics (DOC 39 kb) [file 12885_2016_2692_MOESM2_ESM.doc]

Additional file 2: Table S2. **Relationship between non-aspirin NSAID consumption and breast cancer according to COX2/COX1 selectivity and tumor characteristics**

| **Variable** | **Category** | **NSAID** | **OR (95% CI)** | **p** |
| --- | --- | --- | --- | --- |
| **Clinical stage** | **1-2** | cox1 selective | 0.89 (0.72-1.10) | 0.2734 |
|  |  | cox2 selective | 0.62 (0.43-0.89) | 0.0103 |
|  | **3-4** | cox1 selective | 0.87 (0.59-1.30) | 0.5021 |
|  |  | cox2 selective | 0.83 (0.42-1.62) | 0.5811 |
| **Pathology** | **Ductal cancer** | cox1 selective | 0.77 (0.62- 0.94) | 0.0095 |
|  |  | cox2 selective | 0.64 (0.45-0.91) | 0.0122 |
|  | **Non-ductal cancer** | cox1 selective | 0.90 (0.62-1.31) | 0.5791 |
|  |  | cox2 selective | 0.63 (0.32-1.25) | 0.1857 |
| **Inmunohistochemistry** | **Hormone +** | cox1 selective | 0.79 (0.64-0.98) | 0.0289 |
|  |  | cox2 selective | 0.61 (0.42-0.88) | 0.0082 |
|  | **HER2+** | cox1 selective | 0.64 (0.44-0.93) | 0.0188 |
|  |  | cox2 selective | 0.64 (0.35-1.19) | 0.1569 |
|  | **Triple negative breast cancer** | cox1 selective | 0.97 (0.61-1.49) | 0.8376 |
|  |  | cox2 selective | 0.84 (0.40-1.76) | 0.6472 |

OR: Odds ratio adjusted for age, recruitment area, education level, tobacco smoking history, BMI, family history of breast cancer, number of deliveries, age at first delivery, menarche age, and menopausal status. CI: confidence interval
